# Implantable biomaterials to provide local immunotherapy following surgical resection

**DOI:** 10.18632/oncotarget.26487

**Published:** 2018-12-28

**Authors:** Michael J. Gough, Jason R. Baird, R. Bryan Bell

**Affiliations:** Earle A. Chiles Research Institute, Providence Cancer Institute, Providence Portland Medical Center, Portland, OR, USA

**Keywords:** immunotherapy, head & neck, cancer, surgery, biomaterials

Surgical extirpation of the primary tumor and draining lymph nodes followed by histopathologic risk-adapted adjuvant radiation or chemoradiation remains the standard of care for most patients with Head and Neck Squamous Cell Carcinoma (HNSCC). However, non-viral associated (HPV^−^) HNSCC is characterized by a high rate of therapeutic resistance: approximately 30–50% of these patients have local or distant recurrence following conventional treatment. Furthermore, there is some evidence to suggest that extirpative surgery itself is immunosuppressive and may promote cancer progression [1]. Identification and successful integration of novel immunotherapy into existing treatment paradigms of surgery and radiation for HNSCC is an evolving treatment approach that has the potential to overcome suppressive mechanisms within the tumor and lead to enhanced survival and decreased morbidity for patients with locally advanced or recurrent HNSCC [2]. Our research in preclinical models has shown that immune responses play an important role in local tumor control following surgical resection [3], and there is increasing evidence that the addition of local or systemic immunotherapy before (neoadjuvant) or after (adjuvant) surgery may enhance survival [3–5].

The tumor environment is the primary target site for anti-tumor immune responses, but commonly evolves during malignant progression to include a range of suppressive mechanisms. Information obtained from the surgical resection specimen may be leveraged to tailor immunotherapy interventions targeting the surgical site to eliminate minimal residual disease and minimize recurrence. Biomaterial platforms can be constructed to provide a local delivery system for such immunotherapies, which can be applied to the resection bed at the time of surgery, and be utilized to enhance the effectiveness of surgery. We recently demonstrated that cyclic-di-nucleotides (CDN), which are ligands of STimulator of INterferon Genes (STING), incorporated into a simple biomaterial and placed into the resection cavity were able to eliminate residual disease in preclinical models of HNSCC [4]. CDN are naturally generated following cGAS recognition of cytoplasmic DNA from endogenous sources or following intracellular infection, and CDN binding to STING results in activation of IRF3 and transcription of type I IFN. STING therefore forms part of an endogenous nucleic acid sensing mechanism that can be exploited for cancer therapy [6]. Initial studies using direct injection of CDN into the tumor resulted in CD8 T cell-mediated clearance of cancer cells [7]. We found that application of STING in a biomaterial to the resection cavity prevented recurrence of residual disease [4], consistent with other investigators [5]. The mechanism required host responses to inflammatory cytokines and as with direct injection, the final tumor clearance was mediated by CD8 T cells [4, 5]. These data suggest that biomaterial platforms present an opportunity to orchestrate local immune responses in the surgical site to prevent tumor recurrence. While there is a clear rationale to apply this to prevent HNSCC recurrence, this approach is equally applicable to a range of other malignancies.

HNSCC patients respond variably to conventional cancer therapies, in part because of their differing anti-tumor immune status [8, 9]. Similarly, not all patients respond to immunotherapy combinations, likely for similar reasons. To understand this variability, we developed an ‘explant assay’ using fragments of tumor stimulated *ex vivo* with innate adjuvants [4]. This can also be achieved using single cell suspensions of tumor-infiltrating cells [10], but the explant approach has the advantage of preserving the geographic relationship between the different cell types and their regulatory networks. Importantly, this approach highlighted the diversity of responses to the same agents between different patient tumors. Using this approach, we noted IL-10 production in murine tumors that responded poorly to STING ligands, and that the addition of antibodies that block IL-10 improved the response in these tumors [4]. In this way, analysis of the excised tumor for its biological response to immunotherapy may permit personalization and expand the in vivo response rate.

**Figure 1:**
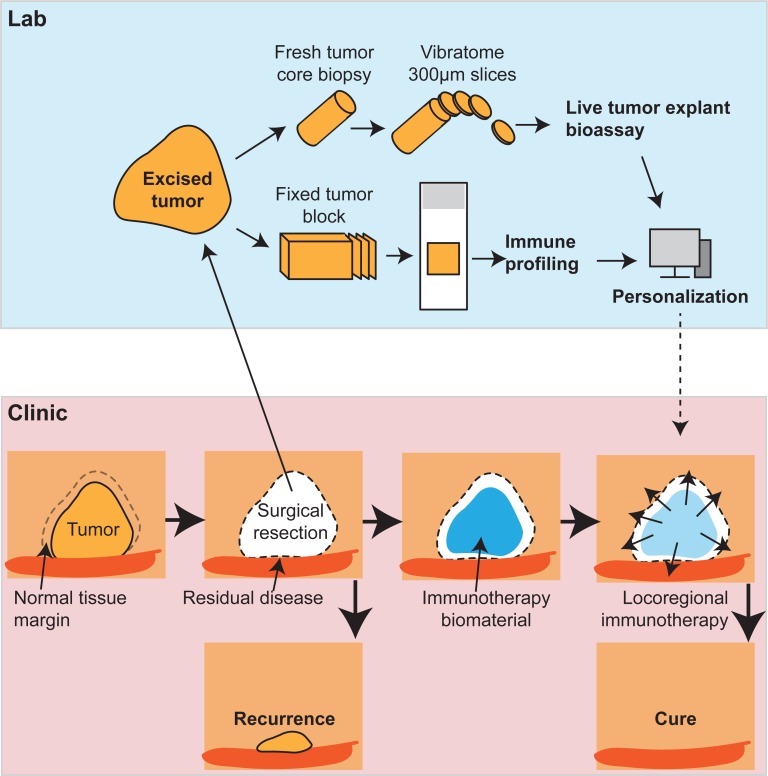
The combination of implantable biomaterials together with *ex vivo* analysis of the tumor immune biology permits personalization of surgical immunotherapy to prevent tumor recurrence

Immunotherapy for cancer is no longer a theory, but along with all other cancer therapies, we need to know why some people respond and others do not. If tumor explants provide an authentic guide to the local response to immunotherapy agents, they can permit rapid screening of patient tumors against multiple agents. More importantly, they may help provide a mechanistic understanding of the key cell types, differentiation status, and geographical relationships that dictate the response to immunotherapy. Together, *ex vivo* analysis of the biological response of the tumor may permit personalization of immunotherapy to generate cures in patients that are currently unresponsive. Finally, understanding the key immunological processes that need to occur at the resection site to control residual disease has the potential to expand the role of surgery beyond cytoreduction and develop it as an immunological event.
